# Physiological routes from intra-uterine seminal contents to advancement of ovulation

**DOI:** 10.1186/1751-0147-48-13

**Published:** 2006-08-03

**Authors:** Dagmar Waberski, Anke Döhring, Florencia Ardón, Nadine Ritter, Holm Zerbe, Hans-Joachim Schuberth, Marion Hewicker-Trautwein, Karl Fritz Weitze, Ronald HF Hunter

**Affiliations:** 1 Unit for Reproductive Medicine of Clinics/Clinic for Swine and Small Ruminants, University of Veterinary Medicine Hannover, Germany; 2 Institute for Reproductive Biology, University of Veterinary Medicine Hannover, Germany; 3 Clinic for Ruminants, University of Munich, Germany; 4 Institute for Immunology, University of Veterinary Medicine Hannover, Germany; 5 Institute for Pathology, University of Veterinary Medicine Hannover, Germany

## Abstract

Whole boar semen or seminal plasma has been demonstrated to advance the time of ovulation in gilts. As a means of clarifying this influence, the contribution of uterine lymphatics and their white cell populations has been examined. After duct visualisation with Evan's blue, lymph was sampled from a mesometrial vessel in eight pre-ovulatory gilts whose uterine lumen was infused simultaneously with whole semen in one ligated horn and saline in the contralateral ligated horn. Lymph was collected from cannulated vessels for periods of up to four hours under general anaesthesia. Thereafter, mesometrial lymph nodes, utero-tubal junction and uterine wall tissues were sampled. The proportion of nucleated cells in the sampled lymph increased towards the end of the collection period, but erythrocytes were found in all instances preventing a meaningful differentiation and identification of leukocytes. Prominent uterine lymph nodes were present in the mesometrium on both sides of the reproductive tract in 7 of 10 gilts. Differences in cellular contents were demonstrated between the side of the tract infused with semen and that infused with saline control. Two of 4 gilts had lower values for CD4 (Cluster Differentiation) and 3 of 6 gilts higher values for MHC II (Major Histocompatibility Complex) markers on the side challenged with semen. In contrast, values remained constant for CD8 but ranged widely for CD18. Immunohistochemical analysis of uterine tissue samples for MHC II+ cells revealed significant differences (P < 0.05) between the control and semen-treated ligated portions of the horns, as well as between the tissue sample of uterine wall and that from the utero-tubal junction, but there were no significant differences for CD4+ cells. It therefore remains plausible that semen-induced cytokines in the uterine lymph undergo counter-current transfer to the ipsilateral ovary and accelerate the final maturation of pre-ovulatory Graafian follicles.

## Background

The process of ovulation has continued to attract attention since the appreciation that it is triggered by a surge of gonadotrophic hormones detectable in the systemic circulation [1-4]. This surge is known to be prompted by either a positive feedback influence of oestradiol from the maturing follicle(s) in so-called spontaneous ovulators or coital stimulation in reflex ovulators [4]. Elevated levels of hypophyseal gonadotrophins in the systemic circulation bind to ovarian tissues during the pre-ovulatory interval [5], but the full spectrum of changes consequent upon such binding remains to be described. Classical features include an increase in bloodflow, a progressive shift in steroid hormone synthesis from oestradiol to progesterone representing the onset of luteinisation, a morphological reorganisation of the granulosa cell layers and dissolution of the basement membrane enabling vascularisation of the granulosa, and a resumption of meiosis in the oocyte(s) destined to be shed into the Fallopian tube(s) (reviewed by Hunter [6]).

In recent years, and largely due to a series of stimulating essays by Espey [7-9], the process of ovulation has been likened to an inflammatory reaction. A major feature of such inflammation is infiltration of different populations of white blood cells into and through the tissues of mature Graafian follicles. Cytokines released from the attracted leukocytes are thought to be critically involved in structural changes of the follicular wall [10,11]. It is not known to what extent migrating leukocytes can act as vectors between different components of the reproductive system. In particular the question arises as to whether leukocyte traffic could offer a potential link between the influence of whole semen or seminal plasma components in the uterus and an acceleration of the events leading up to ovulation.

The domestic pig is traditionally regarded as a spontaneously-ovulating species with a rather precise interval between the gonadotrophin surge and ovulation [1,2,12]. Waberski *et al*. monitored ovulation in gilts by non-invasive ultrasound scanning and noted that seminal plasma or fractions thereof could advance the anticipated time of ovulation by a significant number of hours [13-15]. Ovulation was monitored by non-invasive, ultrasonic scanning. Moreover, in a surgically-prepared model in which semen had access to only one of the uterine horns, an accelerated ovulation could be demonstrated on the infused side with no detectable influence on the contralateral ovary [13,14]. No satisfactory explanation for these exciting results has hitherto been proposed, nor has the fact that advanced ovulation occurred in only a proportion of the treated animals received sufficient consideration. If whole semen or seminal plasma were to induce variable degrees of inflammation in different regions along the uterine mucosa, then it seems entirely possible that downstream events, for example ovulation, could be influenced to a variable extent.

In this preliminary report, we have proceeded from the hypothesis that leukocytic responses to the presence of whole semen or seminal plasma components in the uterus could influence mature Graafian follicles by secretion of cytokines. There is sound experimental evidence for the involvement of diverse cytokine molecules in the events of ovulation in mammals [10,11]. A critical question is the manner of transmission of leukocyte-derived cytokines from the genital tract to the gonad. The aim was to study the potential involvement of lymphatic pathways, appreciating that a counter-current transfer of 'programming information' from uterine lymphatics into ovarian arterial blood would eventually need to be involved.

## Methods

### Animals

Ten hybrid gilts, aged 8–9 months and weighing between 100–110 kg, were used in these experiments. They were housed on straw in covered barns, fed a standard commercial diet of concentrated pellets twice daily, and given free access to drinking water. Oestrous cycles were monitored in the presence of a mature boar and the animals prepared for surgery during a spontaneous period of oestrus.

### Premedication and induction of anaesthesia

An intramuscular injection of azaperone (2 mg/kg; Stresnil^®^, Janssen-Cilag GmbH, Neuss, Germany) was given by way of premedication. Approximately 30 mins later, anaesthesia was induced by intramuscular injection of ketamine (10–15 mg/kg; Ursotamin^®^, WDT, Garbsen, Germany). Thereafter, an indwelling catheter was positioned in a prominent ear vein and anaesthesia deepened by intravenous injection of thiobarbiturate (Thiamylal sodium, 2.5–10 mg/kg; Surital^®^, Pharmacia and Upjohn GmbH, Erlangen, Germany). Cessation of the swallowing reflex indicated the desired depth of anaesthesia. After endotracheal intubation, anaesthesia was maintained using a mixture of oxygen, nitrous oxide and isofluran (Isoflo^®^, Essex, Munich, Germany). The animal was placed in a dorsal recumbent position on the operating table.

### Surgical approach

The abdominal skin was washed and sterilised and then fully draped. Following aseptic procedures, the reproductive tract was exposed *via *a mid-ventral incision and, with the minimum of handling of tissues, the number of mature pre-ovulatory Graafian follicles was recorded for each ovary. Double ligatures of braided 3-0 silk (3-0 Mersilk; Ethicon Ltd, Edinburgh, Scotland) were positioned around each uterine horn approximately 25 cm distal to the utero-tubal junction, anchored in the mesometrium, and used to retain fluid instilled into the uterine lumen. By means of disposable hypodermic syringe and needle, 20 ml of warm, freshly-collected boar semen were introduced into the ligated portion of one horn and 20 ml of warm sterile saline (phosphate-buffered) into the contralateral horn. This volume of fluid did not cause distension of the uterine tissues.

**Figure 1:**
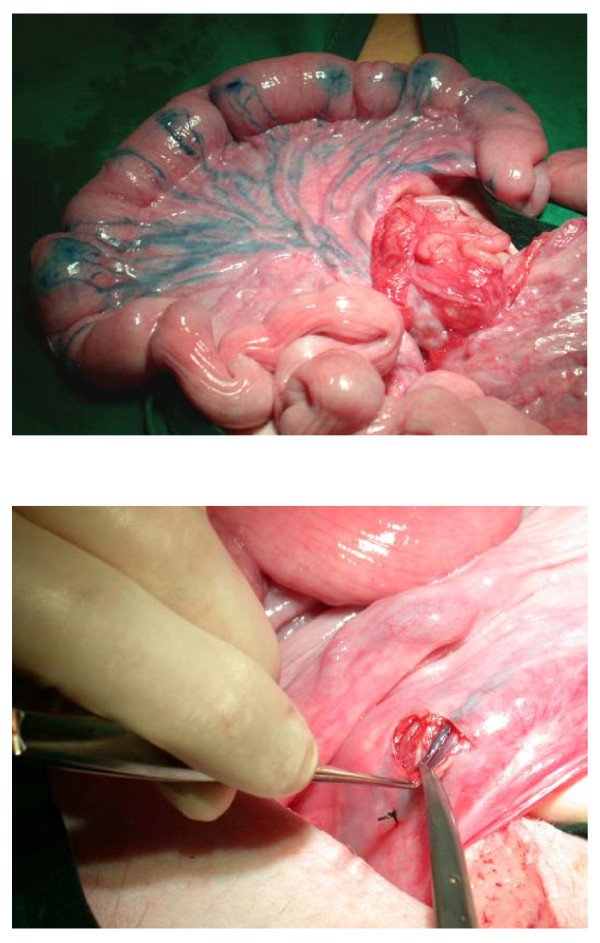
Surgical preparations to demonstrate (A) the visualisation of uterine lymphatic drainage with Evans blue, and (B) that the dissection around a lymph vessel somewhat distended due to a distally-placed fine silk ligature.

In order to visualise lymphatic vessels draining from the uterus, a solution of 0.5% Evans blue in 0.9% saline was introduced under the serosal layer of appropriate regions of each uterine horn using a 1 ml disposable syringe and 26 gauge intradermal needle. The dye was visible in mesometrial lymphatics within a few minutes (Figs 1A,B), enabling the preparation of a prominent vessel for cannulation and collection of fluid. Irridectomy scissors were used to dissect the overlying mesometrium. Fine silk ligatures (4-0, Mersilk; Ethicon Ltd, Edinburgh, Scotland) were positioned around the exposed vessel close to the intended site of cannulation. The downstream ligature was then tightened leading to distension of the lymph vessel, and a tapered polyethylene cannula (int. diameter 0.2 mm, external diameter 0.4 mm, length 8 cm; Fine Science Tools, Foster City, USA) introduced upstream through a small incision made in the wall of the lymph vessel with the tip of the irridectomy scissors. The tip was located between valves and the second ligature tightened.

Some 45–60 mins after introducing semen or saline into the respective uterine horns, lymph flowing along the cannula was collected into an EDTA polyethylene vial. Collection continued for a period of 45 mins and the vial was changed every 15 mins. Reproductive tissues were maintained in a moist condition during collection by means of irrigation with warm saline. After sampling and withdrawal of the cannula, the uterine lymph nodes in the mesometrium were removed by careful blunt dissection, and samples of tissue were also taken from the utero-tubal junction and wall of the ligated portion of the uterus. The tissues bordering these sampling sites were then apposed with sutures of 4-0 Mersilk.

The mid-ventral incision was closed in three separate layers, and animals given a systemic injection of antibiotics (sodium amoxicillin; 10 mg/kg; Gramox^®^, Vetoquinol, Oberursel, Germany). They were returned to recovery pens bedded with straw. There were no detectable post-operative complications.

### Cellular analysis

The number of cells in samples of lymph was counted using a haemocytometer slide. Cells were classified as nucleated (leukocytes) and erythrocytes. As to the lymph nodes, these were cut into small pieces and cells were flushed out with PBS. Lymph node cells were analysed by flow cytometry (FACScan^®^, Becton Dickinson) after staining with monoclonal antibodies specific for CD4, CD8, and MHC II (major histocompatibility complex class II^+^) markers. The antibodies were chosen to monitor changes among T-cell subsets (CD4, CD8) and to assess activation-induced changes (MHC II) of surface antigen expression.

Classical immunohistochemistry methods were used in this study following the protocol of Boenisch [16], with slight modifications as given in Döhring [17]. Tissue samples were either snap-frozen in liquid nitrogen or fixed in formaldehyde and embedded in paraffin wax. After appropriate sectioning, MHC class II and CD4 antigen expression was visualized after sequential incubation with primary monoclonal antibodies (mab H42, anti-MHCII, mab 74-12-4, anti-CD4, both 1:150 in phosphate buffered saline (PBS) with 1% bovine serum albumin (BSA), 22 h, 4°C) and a biotinylated secondary antibody specific for mouse IgG (GAM-6, 1:200 in PBS, 10% pig serum, 30 min). Binding was detected using the avidin-peroxidase complex with biotin as a substrate.

## Results

Ten animals underwent the above procedures and samples of lymph were collected in eight of them.

As anticipated, the flow of lymph was slow (approximately 2.5 ml/hour but varying from 0.9–5.0 ml/hour) and samples were invariably pale pink in colour due to blood contamination. The specific site of contamination was uncertain but was thought to be the small puncture wounds made in the uterine surface during introduction of the Evans blue dye.

**Figure 2:**
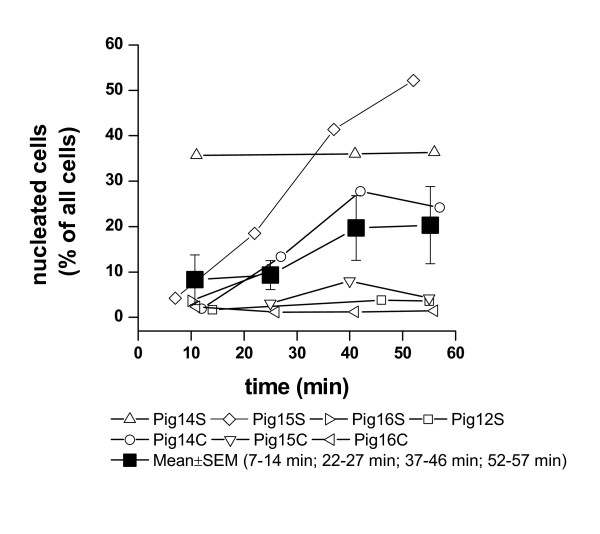
Relative changes in the proportions of leukocytes (nucleated cells) among cellular components (leukocytes and erythrocytes) in uterine lymph collected during a 60-minute sampling period. C: control, uterine horn infused with saline; S: uterine horn infused with semen.

The proportion of nucleated cells in the sampled lymph increased as the collection period lengthened (Fig. 2), but erythrocytes were found in all instances preventing a meaningful differentiation and identification of leukocytes. Accordingly, it was not considered appropriate to perform a sorting of leukocytes.

Immunohistochemical analysis of uterine tissue samples for MHC class II-positive cells revealed significant differences between the control and semen-treated (higher values) horns (Table 1), as well as between the tissue sample of uterine wall and that from the utero-tubal junction (higher values). No significant differences could be observed for CD4-positive cells (data not shown).

**Table 1:** Numbers of MHC class II-positive cells in different locations of uterine tissues after exposure to semen or saline.

		Localization				
Localisation^1^	Exposure	Surface epithelium	Superficial stroma	Deep stroma	Glandular epithelium	Total
			
Uterus (n = 5)	saline	2 ± 1	9 ± 3	8 ± 2	1 ± 0.4	20 ± 6 a
Uterus (n = 5)	semen	3 ± 2	11 ± 1	9 ± 2	1 ± 0.7 x	24 ± 5 b, x
UTJ (n = 4)	saline	3 ± 1 a	12 ± 5	8 ± 3	1 ± 0.2	24 ± 7 a
UTJ (n = 5)	semen	4 ± 1 b	16 ± 1	11 ± 3	2 ± 0.2 y	34 ± 4 b, y

1) UTJ: utero-tubal junction. Data are given as means ± standard deviations (tissue samples from 6 pigs).

a, b: Significance between values with these superscripts in the same location (p= 0.05)

x, y: Significance between values with these superscripts with the same treatment (p= 0.05)

Prominent uterine lymph nodes were present in the mesometrium on both sides of the tract in 7 of 10 gilts. Pair wise comparisons revealed significantly lower percentages of CD4-positive cells in lymph nodes draining the inseminated side (Table 2). Conversely, but not significant, the fraction of MHC class II-positive lymph node cells was higher in such lymph nodes compared to side draining the saline-infused uterus horn. In contrast, values for CD8-positive cells remained constant (data not shown).

**Table 2:** Frequency of CD4- and MHC-class II positive-cells from uterine lymph nodes.

	CD 4-positive cells (%)		MHC class II-positive cells (%)	
Pig No.	Ln S ^1^	Ln C	Ln S	Ln C
11	17	18	60	60
13	37	45	46	37
14	54	60	48	39
15	21	33	44	45
16	n.a.^2^	n.a.	59	60
17	n.a.	n.a.	77	39
	
Mean ± SEM	32 ± 8	39 ± 9	56 ± 5	47 ± 4
paired t-test	0.03		0.10	

1) LN S: lymph node draining the inseminated uterus horn: LN C: lymph node draining the saline-inseminated uterus horn.

2) n.a.: not analyzed due to absence of lymph node.

## Discussion

In terms of clarifying the phenomenon of precocious ovulation in pigs as a response to infusion of whole semen into the uterus, this preliminary study has not provided specific data as to underlying physiological mechanisms. Nonetheless, it has offered one line of guidance which can be developed in future studies when the technical problem of obtaining lymph samples free of blood contamination in acute experiments has been resolved. The relevant observations concern populations of leukocytes and short-term responses to treatments as monitored in uterine tissues and mesometrial lymph nodes.

Considering first the observations on lymph nodes, the CD4 values – lower in 2 of 4 gilts on the side of the tract exposed to semen – do not permit definitive conclusions, nor does the absence of significant differences for CD4+ cells between the two sides in the time frame examined. By contrast, the immunochemical analysis of MHC II+ cells indicated significant differences in uterine tissue samples between the semen-treated and control horns and likewise between regions of the uterus, *ie*. endometrium of (a portion) of uterine horn and the utero-tubal junction. Such differences could demonstrate a rôle of antigen-presenting cells in the immune response following challenge with male antigens. Furthermore, the differing degree of response between uterine tissue and that of the utero-tubal junction could suggest a more sensitive rôle of the junctional region. Important features in the latter regard would be (1) the prompt accumulation of seminal components at the utero-tubal junction due to the influence of powerful myometrial contractions [18-20] and (2) the extremely prominent underlying lymphatic 'vessels' during oestrus [21]. Distension of these lymphatics and thereby of the overlying tissues may function primarily to inhibit free passage of seminal plasma into the Fallopian tube. At the same time, however, lymphatic distension during oestrus would provide a potential route for rapid molecular conversations between seminal contents that bathe the utero-tubal junction and the leukocytic response of the recipient gilt, again supported by the regional differences in the MHC II^+ ^cell response.

Although giving the work an immunological orientation, our original hypothesis remains intact: *ie*. that cytokines secreted by populations of leukocytes responding to the presence of whole semen or seminal plasma components in the uterus could be acting to hasten the process of ovulation in an ipsilateral ovary. The proposals are not completely speculative, for a recent publication implicates endometrial cytokine expression as part of the inflammatory response to seminal plasma with an involvement in the recruitment of leukocytes [22]. As to cytokine activity in the tissues of Graafian follicles, this is now accepted as a classical feature and has been reviewed by Brännström [10], Bukulmez & Arici [11] and Hunter [6].

Cytokines would reach ovarian follicular tissues *via *the ovarian artery, having entered the utero-ovarian artery from uterine lymphatics by means of counter-current exchange [see [23-27]]. The efficiency of such putative counter-current transfer and its proposed influence on ovulation would vary according to the proximity of uterine lymphatic drainage to the utero-ovarian blood vessels, especially the ovarian artery. This is illustrated in the beautiful plastic casts of Gawronska *et al*., [26]. The extent of intimacy between respective lymphatic and blood vessels would influence the effectiveness of counter-current transfer. The stage of oestrus, that is the time before ovulation and thus the prevailing ratio of oestradiol to progesterone, would also influence the counter-current transfer mechanism and the degree of oedema at the utero-tubal junction.

As to potential sites of leukocytic secretion of cytokines induced in response to whole semen or seminal plasma, these would include (1) the massive populations of cells in the uterine lumen infiltrated in response to seminal contact with the endometrium [28], (2) leukocytes situated within uterine mucosal tissues, and (3) leukocytes in lymphatic vessels draining the uterus – or a combination of all three sites. Clearly, the scenario would be dynamic with rapid movement and turnover of different populations of cells as part of an inflammatory response. Variation in the activity of the three preceding routes could account for differing cytokine activity reaching the ipsilateral ovary and a variable influence on the ovulatory response. Indeed, cytokine secretion in the genital tissues could influence populations of leukocytes migrating into the wall of Graafian follicles soon to ovulate and thereby their own local secretion of cytokines. Overall, there would be interactions with a finely-tuned programme of follicular modifications at ovulation.

In a small surgical study of mated pigs (gilts), the presence of semen in the uterus prompted an increase in the concentration of PGF_2α _in uterine venous blood within 15 mins of mating (see [29]; Fig. 3.2). There is little doubt that the counter-current transfer system between utero-ovarian vein and ovarian artery would enable enhanced levels of PGF_2α _to reach an ipsilateral ovary [30-32]. Because the concentration of prostaglandins in the antral fluid of mature Graafian follicles increases dramatically in the hours before ovulation in spontaneously cycling pigs [33], prostaglandin secretion by uterine tissue following mating might also contribute to the process of ovulation by synergising with follicular prostaglandins.

**Figure 3:**
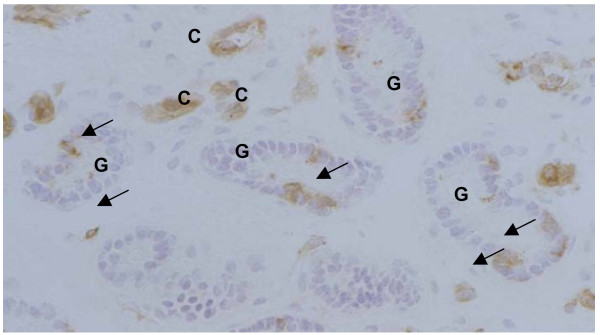
Immunohistochemical staining for MHCII-positive cells. Basal part of the utero-tubal stroma in an inseminated animal. C: Capillaries, G: glands. Arrows indicate MHCII-positive cells in the stroma and glandular epithelium.

Finally, taking into consideration (a) variation between animals; (b) uneven distribution of seminal components between and within the two uterine horns, (c) the degree of distension of individual uterine horns and (d) the precise stage of treatment in relation to the endogenous gonadotrophin surge, then variation in response in a biological endpoint, specifically ovulation, would be anticipated. Concerning the last of these proposals (d), the shorter the interval between the onset of oestrus and ovulation, the lower the probability of demonstrating an ovulatory response to seminal components. This and related temporal aspects are reviewed in Waberski *et al*. [15].

## Conclusion

It remains plausible that semen-induced cytokines in the uterine lymph undergo counter-current transfer to the ipsilateral ovary and accelerate the final maturation of pre-ovulatory Graafian follicles.

## Competing interests

The author(s) declare that they have no competing interests.

**Figure 4:**
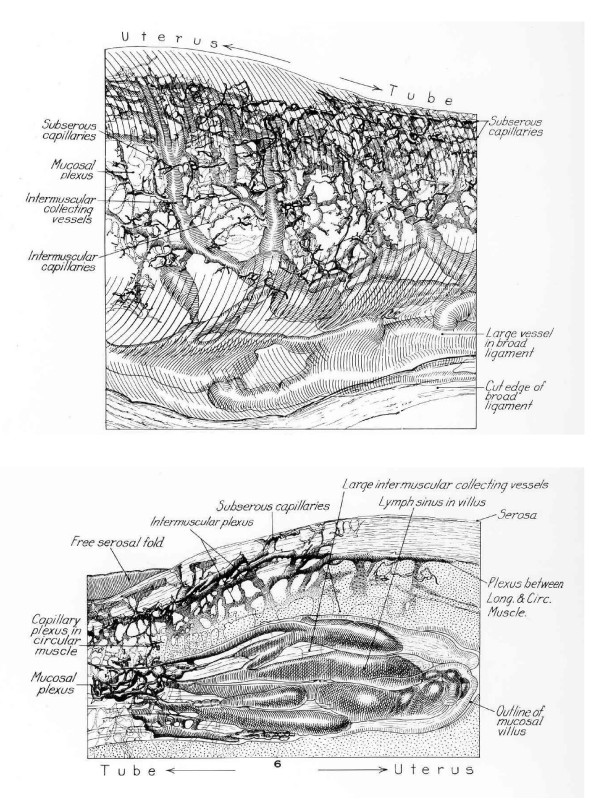
Detailed view of the region of the utero-tubal junction in a domestic pig to emphasize A) the prominent lymphatic vessels that serve to dilate the polypoid processes (B) during oestrus. This arrangement prevents the free passage of fluids – such as semen – from the lumen of the uterus into that of the Fallopian tube and offers a major route for transmission of signals *via *the lymphatic system. (After Andersen 1927)
